# Doctors’ opinion on the contribution of coordination mechanisms to improving clinical coordination between primary and outpatient secondary care in the Catalan national health system

**DOI:** 10.1186/s12913-017-2690-5

**Published:** 2017-12-22

**Authors:** Marta-Beatriz Aller, Ingrid Vargas, Jordi Coderch, Maria-Luisa Vázquez

**Affiliations:** 1 Health Policy and Health Services Research Group; Health Policy Research Unit, Consortium for Health Care and Social Services of Catalonia, Avinguda Tibidabo 21, 08022 Barcelona, Spain; 2 Grup de Recerca en Serveis Sanitaris i Resultats en Salut; Serveis de Salut Integrats Baix Empordà, Hospital, 17-19 Edif. Fleming, 17230 Palamós, Spain

**Keywords:** Clinical coordination mechanisms, Integrated health care, Primary care, Outpatient secondary care, Qualitative research, health personnel’s views

## Abstract

**Background:**

Clinical coordination is considered a health policy priority as its absence can lead to poor quality of care and inefficiency. A key challenge is to identify which strategies should be implemented to improve coordination. The aim is to analyse doctors’ opinions on the contribution of mechanisms to improving clinical coordination between primary and outpatient secondary care and the factors influencing their use.

**Methods:**

A qualitative descriptive study in three healthcare networks of the Catalan national health system. A two-stage theoretical sample was designed: in the first stage, networks with different management models were selected; in the second, primary care (*n* = 26) and secondary care (*n* = 24) doctors. Data were collected using semi-structured interviews. Final sample size was reached by saturation. A thematic content analysis was conducted, segmented by network and care level.

**Results:**

With few differences across networks, doctors identified similar mechanisms contributing to clinical coordination: 1) shared EMR facilitating clinical information transfer and uptake; 2) mechanisms enabling problem-solving communication and agreement on clinical approaches, which varied across networks (joint clinical case conferences, which also promote mutual knowledge and training of primary care doctors; virtual consultations through EMR and email); and 3) referral protocols and use of the telephone facilitating access to secondary care after referrals. Doctors identified organizational (insufficient time, incompatible timetables, design of mechanisms) and professional factors (knowing each other, attitude towards collaboration, concerns over misdiagnosis) that influence the use of mechanisms.

**Discussion:**

Mechanisms that most contribute to clinical coordination are feedback mechanisms, that is those based on mutual adjustment, that allow doctors to exchange information and communicate. Their use might be enhanced by focusing on adequate working conditions, mechanism design and creating conditions that promote mutual knowledge and positive attitudes towards collaboration.

**Electronic supplementary material:**

The online version of this article (10.1186/s12913-017-2690-5) contains supplementary material, which is available to authorized users.

## Background

Increasing medical specialization, rapid medical and technological breakthroughs and the way health care is organized mean that patients see an ever-expanding array of professionals in a variety of different settings [1, 2], thus making clinical coordination difficult and jeopardising the quality and efficiency of health care [1–3]. Concerns over poor clinical coordination have sparked the introduction of a variety of mechanisms to improve it [2, 4].

The most accepted framework to categorize coordination mechanisms is the one initially proposed by March and Simon [5], further developed by Van de Ven and Delbecq [6], Mintzberg [7] and Galbraith [8], and later applied to healthcare coordination mechanisms by Terraza et al. [9]. This framework differentiates between coordination by standardization, which is based on programming, and by feedback, which is based on direct supervision and mutual adjustment [7]. Mechanisms based on programming aim to coordinate by standardizing skills (e.g. ongoing medical training), processes (e.g. shared protocols) or outcomes (e.g. performance monitoring systems) in advance [3, 7, 9]. These mechanisms are considered especially useful for those situations that can be anticipated and do not necessarily require a rapid response [7]. Feedback mechanisms are based on communication and exchange of information between professionals [3], and include direct supervision (e.g. process manager) and mutual adjustment processes, such as the use of communication tools (e.g. telephone, e-mail), shared information systems (e.g. shared electronic medical record) and the use of liaison roles [3, 7, 9]. Mutual adjustment mechanisms achieve clinical coordination through communication or exchange of information between two or more individuals in order to solve the problem at the same level at which the information has been generated [9, 10]. These mechanisms are considered especially useful in those situations where the volume of information to be processed is high and the activities are highly specialized and interdependent [3, 7, 11]. Finally, some mechanisms, such as virtual consultations and joint clinical case conferences between doctors, combine mutual adjustment processes with skills standardization through the training of doctors.

Evidence on the impact of the implementation of mechanisms of coordination between primary and outpatient secondary care comes from effectiveness studies focusing on clinical and services utilization outcomes [2]. Research into the professionals’ perspective is less common, and is more frequently addressed to the evaluation of mechanisms to transfer information across care levels (primary care and secondary care level), including those based on information technology [12–18] and referral forms [19], with little attention paid to other coordination mechanisms. Most of these studies focus on the analysis of factors influencing the uptake of mechanisms in their early implementation [12, 14, 20–22], and scarcely analyse their contribution to clinical coordination or factors influencing their use beyond early implementation. Moreover, few studies explore the overall contribution of available mechanisms to improving clinical coordination [10, 23, 24], since studies generally focus on a single mechanism.

The Spanish national health system is financed by taxes and decentralized into regional health services, with almost universal coverage and free access at point of delivery [25]. The main strategy employed to guarantee clinical coordination across care levels is the assignment of citizens to a primary care team, which acts as gatekeeper and is responsible for coordinating the patient’s care along the care continuum [25]. Secondary care acts as a consultant for primary care and is responsible for more complex care [25]. In the Spanish region of Catalonia, the healthcare system is characterized by a split of the financing and provision functions. Whereas the financing function is responsibility of the Catalan Health Service (CatSalut), the provision of services, which is organised in geographical areas, is the responsibility of a number of providers that establish a relationship (via contracts or agreements) with the CatSalut. These providers are on the one hand, a public company, the Catalan Health Institute, and on the other, public consortia, municipal foundations and private (mostly non-profit) foundations [26], which make up the Integrated Healthcare System for Public Use (Sistema sanitari integral d’utilització pública de Catalunya; SISCAT) [26]. This diversity of providers has generated different management models, including the joint management of both primary and secondary care [27]. Different coordination mechanisms have been implemented by organizations, including shared electronic medical records [27, 28], virtual consultations between doctors [29], clinical guidelines and care pathways [27, 28]. In the context of the Catalan healthcare system, evaluations of the use of the introduced mechanisms are scarce [29–31], and no previous research has explored the contribution of the set of available mechanisms to clinical coordination from the point of view of professionals.

The aim of this article, which forms part of a wider study [32, 33], is to analyse doctors’ opinions on the contribution of mechanisms to improving clinical coordination between primary and outpatient secondary care and the main factors influencing their use in different healthcare settings of the Catalan national health system.

## Methods

### Study design

A qualitative descriptive study was conducted with primary and secondary care doctors.

### Study sample

In a two-stage process, a theoretical or criterion sample was selected, i.e. criteria is defined to ensure that contexts and profiles that could provide information which is different and relevant to the study’s objectives are included [34]. In the *first stage*, three networks were selected to represent the diversity of management models in the Catalan national health system: Baix Empordà county, the city of Girona and the Ciutat Vella district of Barcelona (Table 1). A single entity manages both primary and secondary care in Baix Empordà (Serveis de Salut Integrats Baix Empordà — SSIBE) and in Girona (Institut Català de la Salut — ICS). In Ciutat Vella, two public entities manage primary care (ICS and Institut de Prestacions d’Assistència Mèdica al Personal Municipal — PAMEM) and a different public entity manages secondary care (Parc Salut Mar). SSIBE, PAMEM and Parc Salut Mar are owned by municipal governments, and ICS by the regional government.

**Table 1:** Characteristics of the healthcare networks

	BAIX EMPORDÀ COUNTRY	BARCELONA (CIUTAT VELLA)	CITY OF GIRONA
Population^1^	91,678	99.093	83,312
Location	Rural and semi-urban	Urban	Urban
Primary care providers	SSIBE 4 basic health zones	ICS 4 basic health zones PAMEM 1 basic health zone	ICS 4 basic health zones
Secondary care providers	SSIBE 1 hospital	PSMAR 1 hospital	ICS 1 hospital

*ICS* Institut Català de la Salut, *PAMEM* Institut de Prestacions d ‘Assistència Mèdica al Personal Municipal, *PC* Primary care, *PSMAR* Parc de Salut Mar, *SC* Secondary care, *SSIBE* Serveis de Salut Integrats Baix Empordà

(1) Population ≥ 18 years; Source: Registre Central d’Assegurats (RCA), 2010

All three networks have implemented similar mechanisms for clinical coordination across levels, such as shared clinical guidelines and protocols, virtual consultations between primary and secondary care doctors and joint clinical case conferences. The shared information system implemented, however, differs according to the area: patients attended to in Baix Empordà have a single electronic medical record (EMR) for both care levels, whereas patients attended to in the other two networks have two shared EMR for primary and secondary care [35].

In the *second stage*, in each network primary and secondary care doctors with a minimum of 1.5 years’ work experience in the organization were selected. In order to take in a broad set of experiences, variation criteria were considered during the selection process, taking into account age and sex and speciality of secondary care doctors. To select informants, an institutional contact provided a list of possible candidates according to the above criteria. The sample was selected in a sequential way, so profiles that emerged as relevant in initial interviews were also included in the study. Fifty doctors were invited via email or telephone by their institutions to participate in the study; all of which agreed.

The final sample size was between 15 and 18 doctors per network, and 26 primary care and 24 secondary care doctors in total, which was reached by saturation (Table 2). Half of them were female. The age of the doctors ranged from 34 to 61 years, and their experience in the centres ranged from 1.5 to 35 years. The specialties of secondary care doctors were diverse, and included dermatology, cardiology, emergency care, endocrinology, gastroenterology, internal medicine, nephrology, pulmonology and rehabilitation.

**Table 2:** Characteristics of the doctors’ sample

	BAIX EMPORDÀ		BARCELONA (CIUTAT VELLA)		CITY OF GIRONA	
	PC doctors	SC doctors	PC doctors	SC doctors	PC doctors	SC doctors
Female; male	2; 4	7; 2	ICS: 4; 3 PAMEM: 2; 2	1; 6	6; 3	3; 5
Mean age (range)	51 (44–61)	43 (35–51)	ICS: 49 (34–58) PAMEM: 54 (48–57)	53 (39–60)	50 (39–60)	47 (38–59)
Mean years of experience in centre (range)	16.8 (13–20)	12.5 (1.5–20)	ICS: 14.7 (2.5–21) PAMEM: 14.0 (10–18)	14.2 (1.5–35)	8.6 (1.5–19)	16.7 (7–28)
Medical specialities	Family medicine	Cardiology, emergency care, internal medicine, pulmonology, rehabilitation	Family medicine	Cardiology, gastroenterology, endocrinology, emergency care, internal medicine, nephrology, pulmonology	Family medicine	Cardiology, dermatology, endocrinology, emergencycare, internal medicine

*ICS* Institut Català de la Salut, *PAMEM* Institut de Prestacions d ‘Assistència Mèdica al Personal Municipal, *PC* Primary care, *PSMAR* Parc de Salut Mar, *SC* secondary care

### Data collection

Individual, semi-structured interviews were carried out using a topic guide adapted from previous studies conducted by the research team [10, 31]. The topic guide addressed the professionals’ opinions on the contribution of the available mechanisms to clinical coordination across care levels and the perceived factors influencing their use (Additional file 1).

Interviews were conducted in the healthcare facilities by the first author, an anthropologist and pharmacist with a good knowledge of qualitative methods, the research topic and the context. The interviews lasted between 45 and 80 min and were recorded and transcribed.

Data collection stopped when saturation was reached in each network, which was when encounters with new informants no longer elicited topics which had not been raised by previous informants [36]. Field work took place between July and September 2012 (Baix Empordà) and between December 2013 and May 2014 (Barcelona and Girona).

### Data analysis and quality of information

A thematic content analysis was conducted with the support of the Atlas-ti software. The process of category generation was mainly inductive, i.e. it was oriented towards the identification of emergent patterns in the data [34]. Themes were identified, coded, re-coded and classified, identifying common patterns by looking at regularities, convergences and divergences in data, through a process of constant comparisons, going back and forth between data.

In order to ensure quality of data, the information was triangulated. Results from different groups of informants were contrasted with one another and with the literature. The first author was responsible for the coding and preliminary analysis and worked in close collaboration with the second and last authors, especially in the interpretation and confirmation phases. Differences were discussed and resolved by going back to the data. The analysts had different backgrounds and an in-depth knowledge of qualitative methods, the research topic and the context.

## Results

### Mechanisms contributing to clinical coordination between primary and outpatient secondary care

In all three healthcare networks, doctors identified similar mechanisms that contribute to clinical coordination across care levels in their organizations, mainly feedback mechanisms based on mutual adjustment. Firstly, the shared electronic medical record (EMR) that enables the exchange of clinical information across care levels and contributes to care consistency and patient follow-up across levels. Secondly, mechanisms that enable problem-solving communication and agreement on the clinical approach between primary and secondary care, which varied between networks: in Girona doctors referred to joint clinical case conferences between primary and secondary care doctors, in Barcelona and Baix Empordà to off-line virtual consultations between doctors via EMRs and, in all three networks, to virtual consultations via email. In addition, the institutional telephone emerged as a mechanism that enables to speed up the access to secondary care after referral in urgent cases (Additional file 2). Regarding mechanisms based on programming, referral protocols were perceived to contribute to clinical coordination by facilitating access to secondary care after referral. Other mechanisms, such as clinical guidelines or care pathways, emerged marginally in the discourse of doctors but they did not relate them to clinical coordination.

### Opinions on the mechanisms based on feedback

#### Shared electronic medical record

Doctors of all three networks considered that the EMR allowed them to consult a wide range of clinical data generated at the other care level, helping them to avoid duplicating tests and treatments and to monitor the patient adequately (Table 3, A1). Moreover, as it allowed them to look up the patient’s information, it was easier to resolve queries made via virtual consultations. However, doctors also highlighted the limited recording of information, on the one hand, by primary care doctors on the reason for referral and medical history, and on the other, by secondary care doctors on diagnosis and recommendations. In Barcelona and Girona, secondary care doctors pointed out that the disorganized data entry of primary care doctors made it more difficult to find the information they needed (Table 4, A1).

**Table 3:** Doctors’ opinion on the contribution of coordination mechanisms to clinical coordination in the healthcare networks

Mechanism	Contribution to clinical care coordination	Illustrative verbatim quotes
Feedback mechanisms		
*Shared medical record*	A1) Exchange of clinical information across care levels	“We all have access to the same system and you can access the medical record and look up everything on a patient. Their treatments, the pathologies they have, the last appointments they’ve had, and the medical history are there so you can see more or less what their family doctor envisaged or intended and we can more or less all tow the same line, you know? So you don’t tell them, like, exactly the opposite” (secondary care doctor, Baix Empordà)
*Clinical case conferences between PC and SC doctors*	B1) Rapid resolution of queries	“We go there and we discuss our queries, yeah? Some patients, often we don’t even have to book them in (refer them) any more, we do the things they instruct or advise us to do, and then, later we explain to them how it develops and so there’s no longer any need for an appointment” (primary care doctor, Girona)
	B2) Increases the response capacity of PC doctors	“You also empower the doctor with certain knowledge which gives them confidence with their patients. I think it’s a very important method, I think that purely virtual (…) is great to resolve some cases but, in some cases physically having the doctor in front of you to discuss it is an added element that brings with it all this stuff I’m saying about, let’s say, the empowerment of the doctor” (secondary care doctor, Barcelona)
*Virtual consultations between PC and SC doctors*	C1) Rapid resolution of queries	“It’s fantastic, because if I have a query about a patient, what I used to do was ask about it on the phone and then I’d ask about it by email and now I don’t have to. It’s a job that’s been recorded, the specialist has their own space and I say to them “look, I’m not sure about this patient, he doesn’t seem to be a case for referral. I just wanted you to have a quick look at this and tell me what you think and what we can do”, and this way we save a lot of money, a lot of time and trips” (primary care doctor, Baix Empordà).
	C2) Speed up the diagnostic process	“I’ve used them a couple of times and it went well in the sense that I like to have my patients’ cases all tied up, and when I consulted them, because they solved it for me (…) and he said to me, well, order a Holter for him and if it goes well, discharge, and if not, send him to me for the cardiologist, and he let me order the Holter, because normally they order the Holter, and so they gave me that option” (primary care doctor, Baix Empordà).
*Institutional telephone*	D1) Speeds up access to secondary care	“We answer straight away, we’re delighted to. Look, the other day a doctor who works around here called me, I think he’s one of the switched on ones, with a suspected serious illness, but he called me, eh? And I told him: “Good grief, tell them to come on Thursday”, in two days they had an appointment” (secondary care doctor, Girona).
	D2) Rapid resolution of queries	“They call you (primary care doctors) and say “hi, what’s up, I’ve got this problem”. Well, sometimes they ask: “what should I do? Shall I send him to you or not?” And sometimes, yes, they call you to say “look, I’ve got this patient who’s got this, this and this, and I’m sending him to you”. OK. They even ask me: “what shall I give him in the meantime?”” (Secondary care doctor, Barcelona).
Mechanisms based on programming		
*Rapid diagnostic pathway for cancer*	E1) Speeds up diagnosis and treatment	“If I suspect possible colon cancer, what I do is make an urgent referral to the rapid diagnostic unit (….) And in less than a week they’ve examined that patient, eh? What I mean is yes, our rapid pathway works perfectly” (primary care doctor, Baix Empordà)
*Shared protocols*	F1) Speed up diagnosis and treatment	“(Digestive medicine) has been a service that, well, has taken a long time to get going and, now with these procedures it’s improved, the waiting lists have been managed better. And so we now have the chance to request the diagnostic test directly, if it fulfils the criteria we’ve already agreed on in this protocol” (primary care doctor, Girona)
*Training sessions*	G1) Improve the clinical appropriateness of referrals	“Sometimes in the sessions, for example, urologists have come in and they say “so, patients with prostate problems (…) request analysis, rectal exam, do this, this and that. And then if everything’s fine you don’t have to refer them and the treatment will be this”. Therefore, what they’re giving you is a series of instructions so you can treat the patient but you’re also saving the patient a lot of visits” (primary care doctor, Baix Empordà).

**Table 4:** Doctors’ opinion on problems in the use of available care coordination mechanisms in the studied healthcare networks

Mechanism	Problems in use	Illustrative verbatim quotes
*Shared medical record*	A1) Difficulties in finding the information	“If you see a medical history from a hospital, normally it lists the problems point by point, and normally you’d dedicate a paragraph of, I don’t know, fifteen lines to it. And in the histories from primary sometimes it’s hard to fathom exactly what the problem is with that patient (…) And, I’m telling you, they don’t write much, and it’s not well registered what problems the patients have, you know?” (secondary care doctor, Barcelona).
*Clinical case conferences between PC and SC doctors*	B1) May hinder access to secondary care	“Sometimes they (joint conferences) also complicate access a bit for some patients that you are sure should go to the specialist and until they pass through the filter of the doctor you’re dealing with, well you can’t refer them. And that, in some cases, can take some time” (primary care doctor, Girona).
*Virtual consultations between PC and SC doctors*	C1) Limited description of reason for referral	“You need to spend more time digging around in the ECAP system (EMR in primary care) or unifying it with the IMASIS system (EMR in secondary care), and try to get an overview of the situation. And if in doubt…if we detect that there really is a problem what we ask them to do is refer the patient to us and we end up examining them ourselves properly from top to bottom, and we can clear up all our doubts” (secondary care doctor, Barcelona).
	C2) Don’t check their work email	“I use it sometimes (email), because when, for example, they send you patients to the outpatient department (...) And when for example I reject a..., I reject a request, then I write to the doctor by email (…) and I say “look, I saw your request…” and I explain the reasons for rejecting it, you know…The surprising thing is that no, they don’t open their work email. When I send emails I ask for that thing to confirm if they’ve read it, yeah? And the surprising thing is that they don’t open their email” (secondary care doctor, Girona).
*Institutional telephone*	D1) Difficulties in attending to phone calls	“Maybe an internist calls me. So the admin staff let me know “hey, doctor so and so from Hospital del Mar is on the line”. And I’m in the middle of an appointment and I say, oh dear, if she’s calling something must have happened. And so I have to stop my appointment to talk to her, because if they take a message, maybe when I call her back she’s not there any more, or she’s on duty or whatever. It’s really difficult, isn’t it?” (primary care doctor, Barcelona).

*EMR * electronic medical records

#### Joint clinical case conferences between primary and secondary care doctors

Doctors considered that the joint clinical case conferences mainly conducted in person but also via videoconferencing allowed them to resolve queries about the patient’s care and agree on their clinical approach, enabling faster diagnosis and treatment and avoiding referrals to specialists, which in turn contributes to a reduction in waiting times (Table 3, B1). Due to the interaction produced between doctors, conferences promote a greater knowledge of the skills and resources available in primary care (such as working conditions, diagnostic tests to which they have access), which leads to a better attitude on the part of specialists towards collaboration and more communication outside of meetings (telephone and email). Furthermore, they contribute to improving the training of primary care doctors, which increases their capacity to independently resolve cases similar to those discussed in joint conferences (Table 3, B2).

However, primary care doctors in Girona identified certain limitations. Firstly, they stress their work overload, on having to spend time afterwards requesting tests and recording the agreed approach in the medical record. Secondly, they sometimes have to wait a long time to query the case with a specialist because there is not enough time to discuss all the clinical cases they have, or their meeting is cancelled. Lastly, when they are obliged to discuss cases before referring the patient, the mechanism is perceived as a barrier to accessing secondary care (Table 4, B1).

#### Off-line virtual consultation between primary and secondary care doctors

Firstly, via the EMR, which constitutes the main route by which primary care doctors consult with certain specialists, who decide which cases can be resolved by virtual means and which ones require the patient’s physical presence; and secondly, via email, which is used more sporadically. These consultations allow them to quickly clear up any doubts regarding the clinical approach to take for less complex cases and help to avoid referrals (Table 3, C1). Moreover, those conducted via the EMR allow specialists to prioritize face-to-face consultations with patients and to agree on the tests to be performed in primary care, which saves the patient additional visits to secondary care and speeds up the diagnostic process (Table 3, C2). A few specialists also considered that these consultations contributed to the training of primary care doctors.

In terms of limitations, secondary care doctors pointed out that sometimes primary care doctors make a limited description of the reason for the consultation or fail to include a summary of the most relevant clinical history, so they have to spend more time looking for the information in the EMR (Table 4, C1). Secondary care doctors in Girona pointed out that some primary care doctors do not check their email regularly and do not receive the information they send them (Table 4, C2). Lastly, some secondary care doctors considered that the implementation of virtual consultations via the EMR made it less necessary to participate in joint clinical case conferences.

#### Institutional telephone

Primary care doctors use the telephone when they need to communicate urgently with secondary care doctors, mainly to speed up the access of patients to that level (Table 3, D1), but also to clear up any doubts on the clinical management of the patient, assess the need to refer or decide on the clinical approach until the specialist sees the patient (Table 3, D2). However, doctors pointed out the problems involved in locating the professional from the other level and in conducting a phone call while attending to other patients (Table 4, D1).

### Opinions on the mechanisms based on programming

The *rapid diagnostic pathway for cancer* emerged as a mechanism that facilitates immediate access to diagnosis and treatment for patients with suspected cancer (Table 3, E1). More marginally, other referral protocols also emerged: in Baix Empordà, rapid access pathways to certain specialities, and in Girona, agreements drawn up by doctors during joint clinical case conferences that establish criteria for referral and requesting hospital tests. These mechanisms contribute to improving the appropriateness of referrals to specialists and accessibility to certain specialist tests (Table 3, F1).

*Joint training sessions* were considered by some doctors from Baix Empordà as a mechanism that contributed to improving the clinical appropriateness of referrals because as a result primary care doctors are better trained (Table 3, G1); however, they also pointed out that the number of specialities participating in this type of session is in decline.

### Factors influencing the use of clinical coordination mechanisms

Doctors of both levels identified various organizational and professional factors that influence the use of coordination mechanisms, with some differences between networks.

#### Organizational factors

*Insufficient time to use the mechanisms* emerged as one of the main factors hindering their use in all three networks: this makes it difficult to properly record the details of the visit and look up information in the clinical history, to participate in joint clinical case conferences and follow them up, and to use email or the telephone, in the latter case due to difficulties in locating the doctors of the other care level (Table 5, A1). In Barcelona, the *incompatible timetables* of doctors of different levels emerged as a factor that hinders the organization of joint clinical case conferences and communication by telephone (Table 5, A2). Finally, the *design of the mechanisms* emerged as a determinant of their use. In the shared EMR, for example, the layout of the medical history makes it difficult to organize and quickly identify the information recorded by other professionals (Table 5, A3). With regard to joint clinical case conferences, primary care doctors in Girona pointed out that when they are conducted virtually via videoconferencing, they are less productive because cases are not discussed in such detail and less knowledge is acquired of the skills and resources available in primary care (Table 5, A4). Moreover, sometimes the amount of time scheduled for the conferences is not enough, which prevents the discussion of all the clinical cases presented by the primary care doctors.

**Table 5:** Doctors’ opinion on factors influencing the use of available care coordination mechanisms in the studied healthcare networks

Type of factor	Illustrative verbatim quotes
*Organizational factors*	
A1) Insufficient time to use mechanisms	“Lack of time is what mainly…Of course, you’ve got your job, and this should also form part of the job, shouldn’t it? Being able to do things to help so there’s more communication and more coordination, which we already try to do, you know? But basically I think that (the problem) is lack of time or that this time isn’t included in our working hours, our contracted hours, you know?” (secondary care doctor, Baix Empordà)
A2) Incompatibility of timetables to participate in joint clinical sessions	“Sometimes it’s due to problems that primary care doctors have, because they don’t finish at two, they finish later and don’t have time to get there, and those that start later, at three, well sometimes they can’t get there beforehand to give us time (…) if they can’t find the time to go sometimes it’d be more practical to just refer the patient (…). And then of course the specialists already knew that they tended not to turn up, so normally they would also leave at, when they finished at two, so in the end, between one thing and another the face-to-face consults weren’t very practical” (secondary care doctor, Barcelona)
A3) Design of mechanisms: EMR	“There are a lot of duplicated diagnoses. You’ve got a patient, you send them to emergencies and you send them with a generic diagnosis, maybe “stomach pains”, and maybe it’s appendicitis, for example. The one who sees them in emergencies, and the one who operates on them, doesn’t change the diagnosis and adds a new one instead. And so, when you want to put the medical record in order and you want to drag across the processes to put them all into one and link them with an X-ray, well (the EMR) won’t let you do it because (the episode) is closed. (…) I think that a lot has been implemented but the same old problems haven’t been solved.” (primary care doctor, Baix Empordà)
A4) Design of mechanisms: clinical case conferences	“Well, I suppose that as there’s no real contact (by videoconference), they don’t resolve as many issues. (…) the truth is that I don’t comment on any of them (via videoconferencing). I’d rather they saw the patient, instead of commenting on the plates (image testing results) by videoconference.” (primary care doctor, Girona)
*Professional factors*	
B1) Attitude and interest in collaborating with the other level	“Right, so, I need to request another analysis to check a, a renal function, yeah? [they can consult and modify test requests through the medical record] And these specialists don’t look at it, they don’t look at it because there are filters. Because in the medical record I can, firstly I can filter it and I can remove all the information that isn’t mine. And that’s what the specialists do. I mean, we’ve got a computerised medical record but they tick the filter and they only look at their own medical history and they don’t look, they don’t look at anything else. They’re not interested, right?” (primary care doctor, Baix Empordà)
B2) Knowing each other	“In any case, the good thing I was telling you about is that as they’re normally very accessible, and we have a good relationship with everyone, well sometimes we can ask, or have an informal consultation with this person, with this specialist in particular, to talk about the patient and sort some things out, for example” (primary care doctor, Barcelona)
B3) Lack of awareness of how the mechanisms work	“We designed a rapid pathway for cancer (…). A way, a pathway to say when (they should use it), (…) I also did a session for them, I explained the criteria to them (…), and they carry on sending people via the rapid diagnostic circuit without them fulfilling any of these criteria. I mean, there may be an urgent test required, yeah? I’m not saying there isn’t, but it doesn’t fulfil the criteria to use this pathway” (secondary care doctor, Barcelona)
B4) Concerns over making diagnosis without physical presence of patient	“And the fear is this (on participating in clinical case conferences), that some day there may be something that has to wait and then is made to wait too long, or that the details are lost (…) or (there’s some problem with) transmitting the information” (primary care doctor, Girona).

*PC* Primary care, *SC* Secondary care, *EMR* electronic medical records

#### Professional factors

*The attitude and interest* of professionals in collaborating with doctors from the other level emerged as a factor that influences their recording of information in the EMR and their attendance and participation in joint clinical case conferences (Table 5, B1). The factor of *knowing each other* influences both their level of participation in the conferences and their use of informal communication mechanisms to get support in the management of patients (Table 5, B2). According to secondary care doctors, *lack of awareness of the criteria* for referring patients by virtual means or via the rapid diagnostic pathway for cancer leads some primary care doctors to misuse these mechanisms (Table 5, B3). Lastly, *concerns over making mistakes in diagnosis* due to not presenting cases comprehensively emerged as a factor that affects the participation of some primary care doctors in joint clinical case conferences and influences the specialists’ decision on whether to implement virtual consultations (Table 5, B4).

## Discussion

### Mechanisms that contribute most to clinical coordination are those based on mutual adjustment

Our findings suggest that the main mechanisms contributing to clinical coordination across primary and outpatient secondary care are those based on mutual adjustment processes, which include the shared EMR, joint clinical case conferences between doctors, off-line virtual consultations and the use of telephone for communicating in urgent cases. These mechanisms contribute to clinical coordination by enabling doctors from the two care levels to share information on patient care, as well as to establish direct, problem-solving communication, which translates into improved consistency of care, follow-up and accessibility across care levels. In addition, joint clinical case conferences generate interaction between doctors, which encourages mutual knowledge and positive attitudes towards collaboration, thus promoting the use of other mechanisms.

We expected to find differences in the type of care coordination mechanisms implemented in the networks since they are managed by different providers and represent different management models. However, our findings suggest similar implementation of mechanisms in the three healthcare networks, with a comparable perceived contribution to clinical coordination. This result could be a consequence of the fact that all three healthcare networks form part of a national health system, and thus share many of the contextual factors that guide the implementation of coordination mechanisms: the same care model based on primary care, with a similar system of funding and incentives for professionals and organizations.

There is little research exploring the perceived benefits of shared EMRs across primary and secondary care under routine conditions [17, 18, 37]. The results of this study reveal that doctors perceive that the impact of the shared EMR goes beyond clinical information coordination, as it also has a significant effect on care consistency and patient follow-up across care levels. Furthermore, it improves the functionality of additional coordination mechanisms based on asynchronous communication by helping secondary care doctors to obtain the information they need to respond to off-line virtual consultations, including those made via EMR and email. Interestingly, these benefits were identified both in networks where there is a single EMR for the two care levels and in networks where there are two different but interconnected EMRs. However, several difficulties were also highlighted, expressed in terms of barriers in the use of shared EMRs, which points toward the need to improve EMR functionality to facilitate orderly data entry and maybe to promote doctors’ information technology skills, as suggested by other authors [17], but also reveals some professionals’ limited awareness of the concept that the EMR is a tool for collective use rather than a personal tool [38].

There is little evidence available as yet on joint clinical case conferences between primary and secondary care doctors, with the exception of a few pilot studies [30, 39–41], which consistently associate them with reductions in the number of face-to-face patient consultations and physical examinations carried out in secondary care. Our study identifies additional paths by which this mechanism contributes to clinical coordination, such as creating the kind of professional interaction that promotes mutual knowledge and a better attitude towards collaboration and more communication outside the conferences. Although these conferences are not part of a formal training program, professionals highlight improvements in the training and response capacity of primary care doctors as a consequence of their participation in these conferences [10, 31]. As a result, clinical case conferences emerge as a comprehensive mechanism that promotes clinical coordination across care levels through both mutual adjustment and standardization of professional skills.

Off-line virtual consultations have been garnering more attention in healthcare studies, and have been shown to reduce face-to-face patient consultations in secondary care and waiting times for secondary care [42–44]. In this study, two types of virtual consultations were identified, those conducted via shared EMRs and those via e-mail. The first was perceived to be a formal mechanism to obtain feedback from specialists, and in some cases to enhance the traditional referral process. In contrast, virtual consultations through e-mail were perceived as an informal way to conduct specific curbside consultations. Accordingly, the use of virtual consultations through the EMR was related to different benefits in clinical coordination from those related to consultations via e-mail, including the prevention of inappropriate referrals and the assurance that patients have had all the required diagnostic testing before being referred. The impact of virtual consultations on primary care doctors’ training emerged only marginally in the discourse of some interviewees, despite this being one of the anticipated results of the study [15, 45]. One possible explanation is related to the particular nature of asynchronous communication, which limits the possibility of engaging in the meaningful discussion that is required to promote the learning process.

Finally, among the mechanisms based on process standardization, only referral protocols and agreements emerged as mechanisms that contribute to clinical coordination by facilitating access to secondary care after referral, albeit in a marginal way. Agreements were generally established during the joint conferences, which supports the case for introducing this type of mechanism alongside mechanisms based on direct communication to increase their adaptation and uptake [24]. In doctors’ opinion, other process standardization mechanisms currently available in organizations, such as practical guidelines or clinical pathways [27], do not contribute to clinical coordination, although they are commonly theorised as coordination mechanisms [3, 7]. One possible explanation is the limited uptake of these mechanisms by doctors; however, we cannot rule out the possibility that doctors tend to conceptualise clinical coordination in terms of direct interaction among professionals, but not in terms of distribution of tasks.

### Factors influencing the use of coordination mechanisms

The main barrier reported in the use of coordination mechanisms is insufficient time during consultations (for example, the recording and uptake of information in the EMR) and outside consultations (for example, participating in joint conferences or responding to virtual consultations). This factor, frequently reported in the literature [10, 17, 31, 46], might be even more relevant in the current context of the economic crisis, which has been associated by professionals with an increase in their work overload [35, 47]. Moreover, it should be noted that some of the mechanisms identified involve additional work for primary care doctors, since they retain patients that were previously referred to secondary care doctors [48]. In keeping with previous research [10, 17, 22], the study results highlight the importance of carefully designing mechanisms to properly respond to the clinical coordination needs of doctors and enhance their usability.

Attitude towards collaboration and mutual knowledge have been linked in this and other studies to an improved utilization of coordination mechanisms [23, 24, 49]. Our results reveal that face-to-face joint clinical case conferences are an effective mechanism to encourage a collaborative attitude [10, 50]. This result is in line with the relational coordination framework [11, 51, 52], which proposes that those mechanisms that facilitate the interaction among professionals improve clinical coordination by promoting personal relationships and encouraging a collaborative attitude. Specifically, participating in these conferences may contribute to improving clinical coordination since doctors are more willing to communicate by telephone and email and record information that is specifically addressed to other professionals in the shared EMR. However, an important barrier to using these conferences - and also virtual consultations - is the doctors’ concerns over possible misdiagnosis when patients are not referred in person to secondary care, perhaps due to a lack of awareness of the objectives and scope of these mechanisms.

### Limitations

This study presents certain limitations which must be taken into account in the interpretation of its results and scope. The heterogeneity of the interviewees in terms of certain characteristics, such as medical speciality and type of centre, means that we cannot rule out the possibility that information saturation was not fully reached, although the main arguments are represented in the results. Furthermore, representatives of the organizations participated in the selection process of informants, so they could have introduced a bias towards a more positive discourse. However, the research team also participated in the selection process, discussing the profiles of the informants, and some informants were identified in a sequential way as part of the sampling strategy. Finally, the fact that the field work in one network was performed at a different time as the other two may be the reason for some of the differences observed between the study areas.

## Conclusions

The study results reveal the importance of mechanisms based on mutual adjustment processes to coordinate clinical care across primary and outpatient secondary care. Specifically, the mechanisms that contributed most to clinical coordination were the shared EMR, which allows doctors to share information; joint clinical case conferences and off-line virtual consultations, which enable direct, problem-solving communication; and use of the telephone, which allows primary care doctors to accelerate access to secondary care. In contrast, the contribution of process standardization mechanisms was limited to coordinating access to secondary care through referral criteria. With regard to factors influencing the use of coordination mechanisms, our results highlight the importance of guaranteeing adequate working conditions to enable professionals to use them properly, including allowing enough time to use mechanisms both during and outside consultations. It is also important to ensure that mechanisms are adequately designed to enable their proper use; this includes technical issues in IT-based mechanisms and adequate timetabling in mechanisms requiring direct, face-to-face interaction. Finally, our results also reveal the major impact of relational factors on the use of mechanisms. Therefore, mechanisms that create spaces for direct communication, such as joint clinical case conferences, emerge as useful strategies for improving interpersonal relationships, thus encouraging mutual knowledge and positive attitudes towards collaboration.

## Additional files

Additional file 1: Interview topic guide. (DOCX 14 kb)

Additional file 2: Mechanisms identified by doctors as contributing to clinical coordination between primary and outpatient secondary care in the studied healthcare networks. (DOCX 18 kb)
