# Supplementary material for: Doctors’ opinion on the contribution of coordination mechanisms to improving clinical coordination between primary and outpatient secondary care in the Catalan national health system
Source: BMC Health Serv Res. 2017 Dec 22;17:842. doi: 10.1186/s12913-017-2690-5 (PMC5741878; doi:10.1186/s12913-017-2690-5)
Supplement: Supplementary file 1 — Interview topic guide. (DOCX 14 kb) [file 12913_2017_2690_MOESM1_ESM.docx]

**Supplementary file 1: Interview topic guide**

- Knowledge and opinion on the mechanisms to coordinate clinical care between primary and secondary care
- Opinion on how the mechanisms contribute to clinical coordination across care levels
- Factors (enablers and barriers) of the use of available coordination mechanisms
- Strategies to improve coordination mechanisms
