# Supplementary material for: Doctors’ opinion on the contribution of coordination mechanisms to improving clinical coordination between primary and outpatient secondary care in the Catalan national health system
Source: BMC Health Serv Res. 2017 Dec 22;17:842. doi: 10.1186/s12913-017-2690-5 (PMC5741878; doi:10.1186/s12913-017-2690-5)
Supplement: Supplementary file 2 — Mechanisms identified by doctors as contributing to clinical coordination between primary and outpatient secondary care in the studied healthcare networks. (DOCX 18 kb) [file 12913_2017_2690_MOESM2_ESM.docx]

**SUPPLEMENTARY FILES**

**Sumugat: Mechanisms identified by doctors as contributing to clinical coordination between primary and outpatient secondary care in the studied healthcare networks**

| **Mechanism**  ***Theoretical definition**** | **Healthcare networks** | | |
| --- | --- | --- | --- |
|  | **Baix Empordà** | **Barcelona** | **Girona** |
| ***Feedback mechanisms*** | | | |
| **Shared medical record**  *Electronically-stored health information on a patient that is shared among doctors of PC and SC* | Common to both levels, sharing the same medical history | Different but interconnected  Specialists co-located in same centre as PC use the same medical record as PC doctors | Different but interconnected |
| **Clinical case conferences between PC and SC doctors**  *Joint meetings involving doctors from PC (one or more) and SC for the joint assessment of the patient's diagnosis and clinical approach (tests to be performed, treatment, follow up)* | PC with rehabilitation and endocrinology services  Face-to face meetings in PC centres on a monthly basis | PC with cardiology, urology and endocrinology services (only one PC centre)  Face-to face meetings in PC centres on a monthly basis | PC with gastroenterology, cardiology, endocrinology, nephrology, orthopaedics, urology, and pulmonology services  On a monthly or bimonthly basis  Two modes: 1) face-to-face (digestive medicine, cardiology, endocrinology, nephrology, pulmonology) in PC centres; 2) by videoconference (orthopaedics and urology) |
| **Virtual consultations between PC and SC doctors *via EMR***  *Asynchronous non-face-to-face consultation between PC and SC doctors via EMR* | PC with rheumatology, pulmonology, cardiology, dermatology and rehabilitation services  Recently implemented | PC with nephrology, urology, endocrinology, and dermatology services  In endocrinology: single referral system. The specialist decides whether to hold a virtual or face-to-face consultation. |  |
| **Virtual consultations between PC and SC doctors *via e-mail***  *Asynchronous non-face-to-face consultation between PC and SC doctors via email* | Access to all the doctors’ work email addresses | Access to all the doctors’ work email addresses | Access to all the doctors’ work email addresses |
| **Institutional telephone**  *Synchronous non-face-to-face consultation between PC and SC doctors via institutional telephone* | Telephone in the surgery, with extension numbers for all departments and services | Telephone in the surgery, with extension numbers for all departments and services | Telephone in the surgery, with extension numbers for all departments and services |
| ***Mechanisms based on programming*** | | | |
| **Training sessions**  *Joint meetings involving doctors from PC and SC that address formative aspects related to the clinical management of patients with a specific characteristic or pathology* | PC with dermatology, pulmonology, neurology, rehabilitation, endocrinology, cardiology and urology services  Variable intervals  Conducted in PC centres |  |  |
| **Rapid diagnostic pathway for cancer**  *Referral protocol designed by the national health system to guarantee patients rapid access to SC when certain types of cancer are suspected* | Rapid diagnostic pathway for cancer | Rapid diagnostic pathway for cancer | Rapid diagnostic pathway for cancer |
| **Referral protocols**  *A tool that guides referral processes among the care levels and includes the definition of clinical criteria* | Existence of referral criteria from PC to urology, dermatology and rehabilitation |  | Shared care protocols (between PC and orthopaedics, rheumatology, rehabilitation and gastroenterology) for standardization of complementary tests and referral criteria. |

PC: Primary care; SC: Secondary care

(*) Authors’ definition based on the literature
